# Neuroprotective γ-Pyrones from *Fusarium Solani* JS-0169: Cell-Based Identification of Active Compounds and an Informatics Approach to Predict the Mechanism of Action

**DOI:** 10.3390/biom10010091

**Published:** 2020-01-06

**Authors:** Hyun Gyu Choi, Ji Hoon Song, Musun Park, Soonok Kim, Chang-Eop Kim, Ki Sung Kang, Sang Hee Shim

**Affiliations:** 1 College of Pharmacy, Duksung Women’s University, Seoul 01369, Korea; movehg@gmail.com; 2 College of Korean Medicine, Gachon University, Seongnam 13120, Korea; jhsong.john@gmail.com; 3 Department of Physiology, College of Korean Medicine, Gachon University, Seongnam 13120, Korea; bmusun1@gmail.com (M.P.); eopchang@gachon.ac.kr (C.-E.K.); 4 Biological Resources Assessment Division, National Institute of Biological Resources, Incheon 22689, Korea; sokim90@korea.kr

**Keywords:** neuroprotection, *Fusarium solani*, endophyte, γ-pyrones, informatics approach

## Abstract

Glutamate toxicity has been implicated in neuronal cell death in both acute CNS injury and in chronic diseases. In our search for neuroprotective agents obtained from natural sources that inhibit glutamate toxicity, an endophytic fungus, *Fusarium solani* JS-0169 isolated from the leaves of *Morus alba*, was found to show potent inhibitory activity. Chemical investigation of the cultures of the fungus JS-0169 afforded isolation of six compounds, including one new γ-pyrone (**1**), a known γ-pyrone, fusarester D (**2**), and four known naphthoquinones: karuquinone B (**3**), javanicin (**4**), solaniol (**5**), and fusarubin (**6**). To identify the protective effects of the isolated compounds (**1**–**6**), we assessed their inhibitory effect against glutamate-induced cytotoxicity in HT22 cells. Among the isolates, compound **6** showed significant neuroprotective activity on glutamate-mediated HT22 cell death. In addition, the informatics approach using in silico systems pharmacology identified that compound **6** may exert its neuroprotective effect by controlling the amount of ubiquinone. The results suggest that the metabolites produced by the endophyte *Fusarium solani* JS-0169 might be related to the neuroprotective activity of its host plant, *M. alba*.

## 1. Introduction

Glutamate is a neurotransmitter that plays pivotal roles in various physiological as well as pathological brain functions. Excessive amounts of glutamate induce oxidative stress-mediated neuronal cell death through both acute brain injuries and neurodegenerative diseases [1]. Reactive oxygen species (ROS) are the major causes of neuronal cell death in chronic neurodegenerative diseases [2]. Earlier studies have shown that excessive release of glutamate in the extracellular environment blocks cysteine uptake and thus depletes intracellular glutathione (GSH) levels, ultimately leading to an accumulation of ROS intracellularly [3]. The use of natural antioxidative phytochemicals for scavenging the free radicals and maintaining homeostasis contributes to alleviation of neuronal cell death [4]. Typically, natural compounds are multiple-target molecules found mainly in microorganisms and plants which exert strong antioxidant activity [5]. Phenolic compounds from the natural sources also exhibit various beneficial effects in cancer, inflammation, and neurodegenerative disorders [5]. This broad spectrum of biological and pharmacological activities has made phytochemicals a suitable candidate for treating multifactorial diseases, such as neurodegenerative diseases.

**Figure 1:**
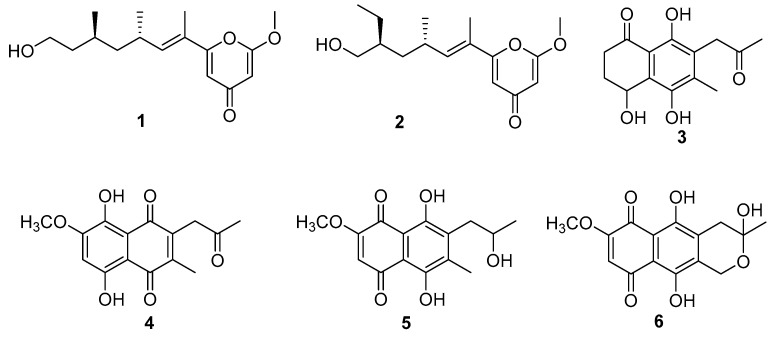
Chemical structures of compounds **1**–**6**.

During our ongoing effort to discover novel bioactive compounds from endophytic microorganisms, we isolated *Fusarium solani* JS-0169 from the leaves of *Morus alba*, which is usually cultivated in Korea, China, and Japan. The leaves of this plant have been traditionally used to feed silkworms to produce silk. This plant has been used for ethnopharmacological purposes to treat many conditions such as asthma, cough, bronchitis, edema, insomnia, wounds, diabetes, influenza, eye infections, and nosebleeds [6]. Recent studies have found that *M. alba* and similar species have cholesterol-lowering [7], anti-viral [8], anti-oxidative [9], and anti-hypotensive activities [10]. *Morus* sp. have been known to comprise prenylated arylbenzofuran and flavonoids, which show neuroprotective effects on glutamate-induced oxidative injury in the HT22 hippocampal cells [11]. Since this host plant has a long history of usage in treatment of various diseases, we hypothesized that its endophytic microorganisms might produce new bioactive secondary metabolites, which may possess similar potential. In this study, we isolated a new γ-pyrone derivative and five known polyketides from cultures of an endophytic fungi *F. solani* JS-0169 (Figure 1). The genus *Fusarium* is widely distributed in geographical and climatic conditions and shows interesting interactions with its host plants. For example, *F. redolens*, isolated from the Himalayan yew, has been reported to produce taxol, an anticancer agent that was originally isolated from barks of the yew tree [12]. Among the secondary metabolites produced by *Fusarium* sp., bikaverin, isolated from *F. oxysporum* f. sp. *lycopersici*, is reported to have potent neuroprotective effects on oxidative stress and anti-apoptotic mechanism to attenuate hydrogen peroxide (H_2_O_2_)-induced neurotoxicity in human neuroblastoma SH-SY5Y cells [13]. The isolated compounds **3**–**6** in this study have a structural similarity to bikaverin, a naturally occurring naphthoquinone-type pigment. Since the host plant extracts show neuroprotective effects and the compounds isolated from cultures of the endophyte have a structural similarity to the known neuroprotective agent, we investigated the potential neuroprotective activity of the isolated compounds on the cultures of *F. solani* JS-0169, subjected to glutamate-mediated HT22 cell death.

In addition, we tried to verify the neuroprotective activities of fusarubin, using the informatics approach. Systems pharmacology has emerged as an approach for identifying the systems-level mechanisms of natural compounds [14,15]. Systems pharmacology can predict the genes that interact with the natural compound based on artificial intelligence, and propose mechanisms of action of the natural compound at the systems-level by exploring gene-related diseases or biological pathways [16,17]. We carried out an additional in silico study to support the hypothesized neuroprotective activity of fusarubin.

## 2. Materials and Methods

### 2.1. General Experimental Procedures

The high-resolution fast atom bombardment mass spectrometry (HRFABMS) data were obtained using a gas chromatography/high-resolution mass spectrometer (JMS-700, Jeol, Tokyo, Japan). The nuclear magnetic resonance (NMR) spectra were acquired with a 300 Ultra shield spectrometer (^1^H, 300 MHz; ^13^C, 75 MHz, Bruker) and an NMR system 500 MHz (^1^H, 500 MHz; ^13^C, 125 MHz, Varian, Palo Alto, CA, USA), using the solvent signals (δ_H_ 7.24/δ_C_ 77.00 for CDCl_3_; Cambridge Isotope Laboratories, Inc., Tewksbury, MA, USA) as internal standards, while chemical shifts were indicated as δ values. Column chromatography was performed over silica gel 60 (70−230 mesh, Merck, Darmstadt, Germany). Silica gel 60 F254 and RP-18 F254S plates (Merck, Darmstadt, Germany) were used for analysis by thin-layer chromatography (TLC) under the detection of ultraviolet (UV) and 10% H_2_SO_4_ reagent to visualize the compounds. The analytical grade of solvents was used for the entire experiments.

### 2.2. Fungal Materials

The fungus JS-0169 was isolated from a leaf tissue of *Morus alba* which was collected from a hill at Choongju, Choongbuk, South Korea in May, 2011. Isolation was conducted according to the previously reported protocol (REF), except for using leaf tissues cut into small pieces (0.5 × 0.5 cm). The fungal strain was identified to be *Fusarium solani* based on conidia morphology and molecular phylogenetics using sequences of translation elongation factor 1-α by Dr. Soonok Kim, one of the authors. The fungal strain was deposited on 20% aq glycerol stock in a liquid N_2_ tank at the Wildlife Genetic Resources Bank (NIBRGR0000114447) of the National Institute of Biological Resources (Incheon, Korea).

### 2.3. Extraction and Isolations

The ethyl acetate (EtOAc) extract (764.7 mg) was subjected to the vacuum liquid chromatography (VLC, 10 × 8 cm) over silica gel using stepwise gradient solvents of hexanes/ethyl acetate (10:0, 9:1. 8:2, 7:3, 6:4. 5:5, 0:10; each 1 L), and 100% methanol (MeOH) (1 L) to obtain ten fractions (fractions 169A-169J). Fraction 169I (188.6 mg) was separated using sephadex LH-20 (50 × 2 cm) with elution of 100% MeOH to obtain compound **6** (5.4 mg) and three sub-fractions (169FA-FC). Afterwards, 169FA (28.7 mg) was purified by using reversed-phase high-performance liquid chromatography (HPLC) (Lunar C18, 5 μm, 250 × 10.0 mm, room temperature, 6 mL/min, H_2_O/ACN, 30:70→70:30, 60 min, UV 210 nm, Phenomenex) to yield compounds **1** (1.2 mg, tR = 34.7 min) and **2** (5.6 mg, tR = 37.5 min). 169G (174.0 mg) was further purified using preparative HPLC with a gradient of acetonitrile/water (ACN/H_2_O) (Lunar C18, 5 μm, 250 × 10.0 mm, room temperature, 6 mL/min, H_2_O/ACN 40:60→60:40, 60 min, UV 210 nm, Phenomenex) to obtain compounds **3** (1.2 mg, tR = 46.1 min), **4** (2.2 mg, tR = 54.9 min), and **5** (15.5 mg, tR = 56.8 min), respectively.

#### 6-((*9R,11R,E*)-13-hydroxy-9,11-dimethyloct-7-en-7-yl)-2-methoxy-4*H*-pyran-4-one (**1**)

Colorless oil. [α]^25^_D_ + 10.9 (*c* 0.1, MeOH); ^1^H NMR, δ_H_ (m, *J* in Hz) 6.21 (1H, d, *J* = 10 Hz, H-8), 6.14 (1H, d, *J* = 1.8 Hz, H-5), 5.47 (1H, d, *J* = 1.8 Hz, H-3), 1.88 (3H, s, H-7′), 3.88 (3H, s, OCH_3_), 2.67 (1H, m, H-9), 1.00 (3H, d, *J* = 6.6 Hz, H_3_-9′), 1.35 (1H, m, H_2_-10a), 1.22 (1H, m, H_2_-10b), 1.58 (1H, m, H-11), 0.90 (3H, d, *J* = 6.5 Hz, H_3_-11′), 1.62 (1H, m, H_2_-12a), 1.36 (1H, m, H_2_-12b), 3.66 (2H, m, H_2_-13); ^13^C NMR, 167.9 (C-2), 89.6 (C-3), 182.2 (C-4), 109.3 (C-5), 160.9 (C-6), 124.1 (C-7), 12.1 (C-7′), 141.6 (C-8), 30.7 (C-9), 20.1 (C-9′), 44.5 (C-10), 27.4 (C-11), 19.8 (C-11′), 39.9 (C-12), 60.9 (C-13), 56.2 (OCH_3_); heteronuclear multiple bond correlation (HMBC) (CDCl_3_, H-#→C-#), H-3→C-2, C-4, and C-5; H-5→C-3, C-4, C-6, and, C-7; H-7′→C-6, C-7, and C-8; H-8→C-6, C-7′, and C-9; H-9→C-7, C-8, C-9′, and C-10; H-9′→C-8, C-9, and C-10; H-10a, 10b→C-11, and C-12; H-11→C-11′, C-12, and C-13; H-12→C-10, C-11, C-11′, and C-13; H-13→C-11, and C-12; (+) high-resolution electrospray ionization mass spectroscopy (HRESIMS) obsd *m*/*z*, 303.1570 [M − H_2_O + H]^+^, calcd for C_10_H_19_O_3_, 303.1567.

### 2.4. 1-diphenyl-2-picrylhydrazyl (DPPH) Radical Scavenging Activity

Each concentrations of the compounds were mixed with an equal volume of 2,2-diphenyl-1-picrylhydrazyl (DPPH) solution (Sigma, St. Louis, MO, USA). After incubation for 30 min at 25 °C, the absorbance value of each compound was measured at 540 nm using an E-Max microplate reader (Molecular Devices, Sunnyvale, CA, USA).

### 2.5. Cell Culture and Treatment.

A murine hippocampal cell line, HT22 cells, was grown in Dulbecco’s modified Eagle’s medium (DMEM; Corning, Manassas, VA, USA) supplemented with 10% fetal bovine serum (Atlas, Fort Collins, CO, USA) and antibiotics (streptomycin/ penicillin; Gibco, Grand Island, NY, USA). HT22 cells were maintained at 37 °C in a humidified incubator supplied with 5% CO_2_.

### 2.6. Measurement of Cell Viability

To assess the cell viability, we used the 3-[4,5-dimethylthiazol-2-yl]-2,5 diphenyl tetrazolium bromide (MTT) assay kit (EZ-CyTox; Daeil Lab Service, Seoul, Korea). HT22 cells were plated on 96-well plates at a density of 1 × 10^4^ per well and incubated for 24 h to adhere. The cells were then exposed to 5 mM glutamate for 24 h with the indicated concentrations of compounds. Cells were then added with 10 µL of Ez-CyTox reagent, followed by incubation for another 30 min. Absorbance values for each compound at 450 nm were obtained using an E-Max microplate reader. The viability of cells was represented by comparing against the percentage of viability of the control group.

### 2.7. Systems Pharmacological Analysis

Systems-level mechanism of neuroprotective action of fusarubin was investigated using the systems pharmacological approach. First, the targets of fusarubin were predicted using the bioinformatics analysis tool for molecular mechanism of traditional Chinese medicine (BATMAN-TCM) platform, which adopts a similarity-based target prediction method (http://bionet.ncpsb.org/batman-tcm) [18]. BATMAN-TCM provides target prediction results for compounds based on various information such as chemical structures, side-effects, ATC (Anatomical, Therapeutic and Chemical) classification system, drug-induced gene expression results, text mining scores, protein sequences, and closeness in a protein interaction network and Gene Ontology (GO) functional annotation. BATMAN-TCM uses the above factors to calculate and displays the probability score of combining the target compound with the protein. The targets were defined as having prediction scores of more than 5. To investigate the systems-level effect of fusarubin, gene set enrichment analysis (GSEA) was performed for predicted target genes using Enrichr platform (https://amp.pharm.mssm.edu/Enrichr/) [19,20,21]. GSEA pathway analysis was performed using the Kyoto encyclopedia of genes and genomes (KEGG) database [22], and the scores were sorted in descending order using the combined score. Target gene-related pathways were defined as having at least two genes involved and *p* < 0.05.

### 2.8. Molecular Docking

The binding between NAD(P)H:quinone oxidoreductase 1 (NQO1) protein and fusarubin was estimated by using molecular docking. The structure of NQO1 was obtained from Protein Data Bank (PDB ID: 1H69) [23], and the structure of fusarubin was extracted from the PubChem database (Pubchem CID: 73421) [24]. The receptor and ligand files were represented in protein data bank (PDB) file format, and standard database format (SDF) file format, respectively. Binding affinity was predicted using Kdeep [25], a 3D-convolutional neural network-based framework. The NQO1 protein was titrated in PH 7.4 by adding protein hydrogens and protonating ligands. The equilibrium dissociation constant (K_d_) and interaction energy between fusarubin and the NQO1 protein were expressed as –log K_d_ (pK_d_) and affinity (kcal/mol).

## 3. Results

### 3.1. Identification of Compounds ***1***–***6***

An endophytic fungus, *Fusarium solani* JS-0169 was isolated from the leaves of a medicinal plant *M. alba*. Chemical investigation of the endophyte JS-0169 afforded isolation of a new γ-pyrone derivative (**1**) and five known compounds (**2**–**6**). Their chemical structures were elucidated by extensive NMR analysis (Supporting Materials). The chemical structures of four known compounds were identified as fusarester D (**2**) [26], karuquinone B (**3**) [27], javanicin (**4**) [28], solaniol (**5**) [29], and fusarubin (**6**) [30] by comparing with the published spectroscopic data (Figure 1).

**Figure 2:**
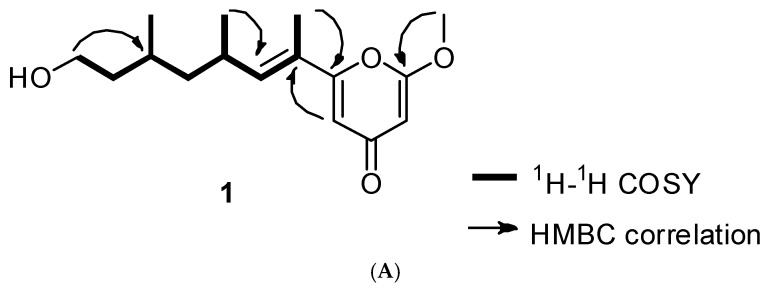

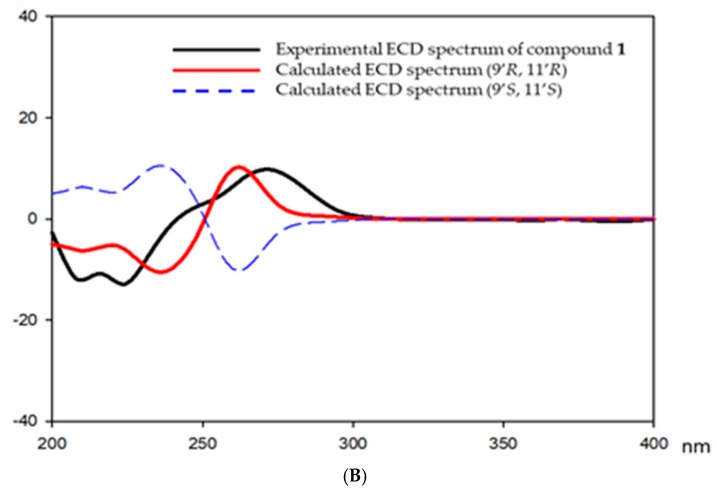
^1^H-^1^H correlation spectroscopy (COSY) and Key heteronuclear multiple bond correlation (HMBC) correlations for **1** (**A**). Comparison of experimental and calculated electronic circular dichroism (ECD) spectra for **1** (**B**).

Compound **1** was obtained as a colorless oil. The molecular formula of **1** was found to be C_16_H_24_O_4_ on the basis of (+) HR-ESI-MS (obsd. [M + Na]^+^ at *m*/*z* 303.1570). The ^1^H-NMR spectrum of compound **1** indicated the presence of the olefinic protons (6.21, 6.14, and 5.47 ppm), an oxymethylene group (3.66 ppm), six aliphatic signals (2.67, 1.62, 1.58, 1.36, 1.35, and 1.22 ppm), three methyl groups (1.88, 1.00, and 0.90 ppm), and a methoxy group (3.88 ppm). The ^13^C-NMR spectrum showed the presence of a ketone C-signal (182.2 ppm), two oxygenated olefinic carbons (160.9 and 167.9 ppm), two olefinic carbons (109.3 and 89.6 ppm), an oxygenated methane carbon (60.9 ppm), a methoxy carbon (56.2 ppm), four aliphatic methylene and methane carbons (44.5, 39.9, 30.7, and 27.4 ppm), and three methyl carbon signals (20.1, 19.8, and 12.1 ppm). Based on the ^1^H and ^13^C NMR data, this compound was found to have a *γ*-pyrone moiety. Strong HMBC correlations of two olefinic proton at δ_H_ 6.14 and 5.47 with the carbonyl carbon at δ_C_ 182.2 ppm supported the presence of *γ*-pyrone moiety (Figure 2A). In addition, the presence of a long aliphatic chain, OCH_2_-CH_2_-CH(CH_3_)-CH_2_-CH(CH_3_)-CH=C(CH_3_), was confirmed by using the ^1^H-^1^H correlation spectroscopy (COSY) spectrum (Figure 2B). The methoxy group was found to be at C-2 of the γ-pyrone ring by HMBC correlation of the methoxyl proton at δ_H_ 3.88 with the oxygenated olefinic carbon at δ_C_ 167.9. The olefinic proton H-5 at δ_H_ 6.14 of the pyrone ring strongly correlated with the olefinic carbon C-7 at δ_C_ 124.1 in HMBC, which suggested that the aliphatic chain was attached to C-6 of the γ-pyrone moiety. It was also confirmed by HMBC correlation of the methyl H_3_-7′ of the aliphatic chain with C-6 of the γ-pyrone ring. Thus, the planar structure of **1** was elucidated as 6-(13-hydroxy-9,11-dimethyloct-7-en-7-yl)-2-methoxy-4*H*-pyran-4-one. The configuration of the double bond in the aliphatic chain was proposed by nuclear Overhauser enhancement spectroscopy (NOESY) correlation. A strong NOESY correlation between H_3_-7′ and H_3_-9′ suggested *E* configuration of the double bond. In addition, a NOESY correlation between the methyl group (H_3_-9′) and a methane proton (H-11) indicates that the two methyl groups, H_3_-9′ and H_3_-11′, were in the anti-orientation. Absolute configurations of C-9 and C-11 were determined by comparing the experimental electronic circular dichroism (ECD) spectrum with the calculated ECD spectrum. Both of them suggested to have *R* configurations since an experimental ECD curve of **1** showed similar ECD cotton effects to those of a calculated one with 9′*R* and 11′*R*. Thus, compound **1** was determined as 6-((*9′R,11′R,E*)-13-hydroxy-9,11-dimethyloct-7-en-7-yl)-2-methoxy-4*H*-pyran-4-one. Except for the splitting pattern of methyl signal in compound **2**, the other ^1^H-NMR data were similar to those of compound **1**. In particular, triplet pattern of methyl signal in compound **2** indicated that the structure of the terminal methyl position was different. The NMR spectroscopic and ECD data were in agreement with the reported data for fusarester D [19]. Thus, the structure of **2** was identified as fusarester D.

### 3.2. Identification of Active Compound and Informatics Approach to Predict the Mechanism of Action

**Figure 3:**
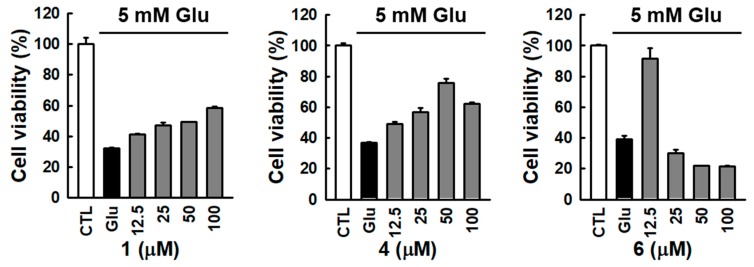
Comparison of protection effect of isolated compounds against glutamate-induced HT22 cell death. Bars denote the percentage of cell viability (mean ± S.E.M).

The neuroprotective effects of the isolated compounds **1**–**6** against glutamate-induced cytotoxicity were evaluated by using a cell viability assay on glutamate-induced HT22 murine hippocampal neuronal cell death. The exposure to 5 mM glutamate reduced the overall cell viability, while the treatment with compounds **1**, **4**, and **6** restored it in a dose-dependent manner (Figure 3). Among them, treatment with compound 6 at the concentration of 12.5 μM significantly increased the cell viability to 90.7 ± 4.5%. Since the effect of compound **6** could be seen at lower concentrations, an additional neuronal cell protection effect was tested at a concentration lower than 12.5 μM.

**Figure 4:**
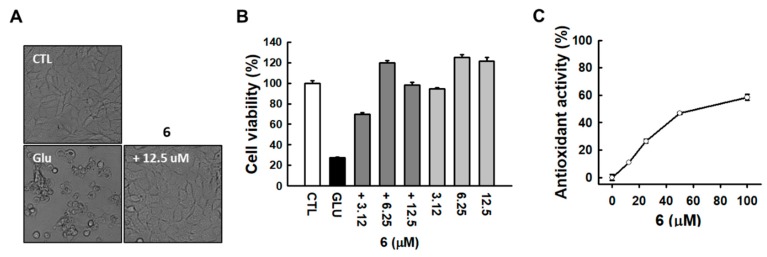
Compound 6 prevented glutamate-induced HT22 cell death. (**A**) Microscopic images were obtained after exposure of HT22 cells to glutamate for 24 h (scale bar, 50 μm). (**B**) Cell viability was measured using a CyTox assay kit 24 h after the treatment with 5 mM glutamate with or without tetrahydrocannabinol (THC). Bars denote the percentage of cell viability (mean ± S.E.M). (**C**) DPPH scavenging activity was determined based on the elimination of DPPH radicals.

As shown in Figure 4A, microscopic images revealed that compound **6** strongly prevented glutamate-induced HT22 cell death. HT22 cells treated with 12.5 μM compound **6** shared a similar morphology with the normal control cells compared to glutamate-treated cells (Figure 4A). The neuroprotective effect of compound **6** was tested at a concentration lower than 12.5 μM, showing sufficient protection at 3.12 μM concentration (Figure 4B). We then tested the antioxidant activities of compound **6** using a DPPH-scavenging activity assay. The results showed that compound **6** exhibited a strong antioxidant property, as indicated by DPPH radical scavenging activity (Figure 4C).

**Figure 5:**
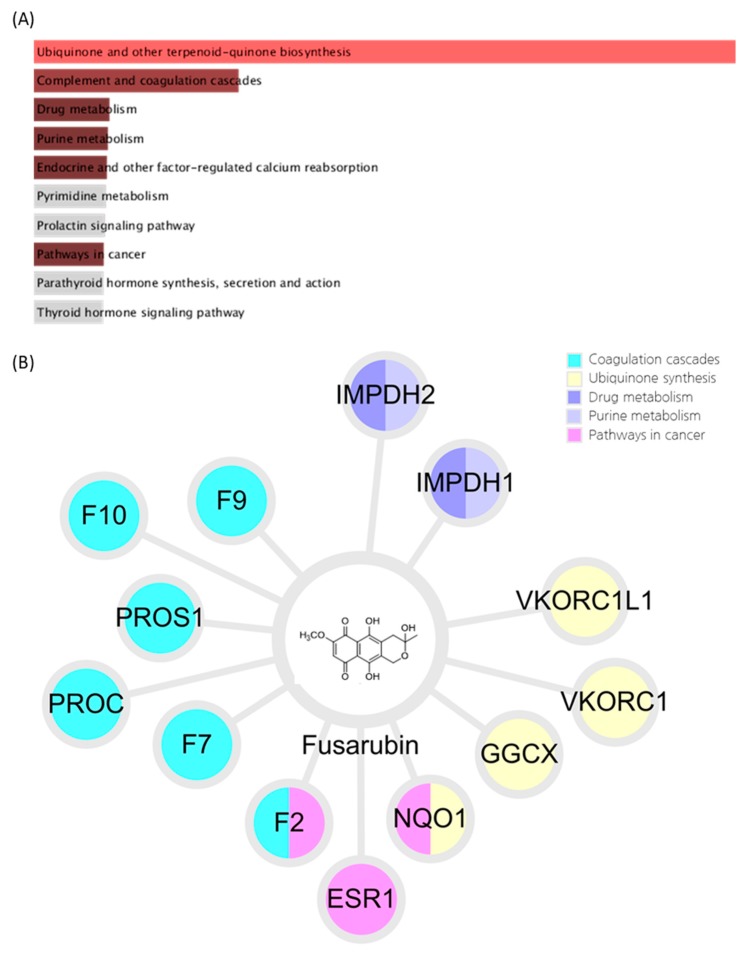
Results of fusarubin analysis using a systems-pharmacological approach (**A**) Pathways related to the predicted targets of fusarubin. Bar graphs represent the combined scores of KEGG pathways calculated using gene set enrichment analysis. The red and brown colored bar graphs represent pathways that have a significant *p*-value. (**B**) Compound-target network of fusarubin. Colored nodes represent the target genes, and each color indicates related pathway with significant combined score. F9, Coagulation factor IX; F10, Coagulation factor X; PROS1, Vitamin K-dependent protein S; PROC, Vitamin K-dependent protein C; F7, Coagulation factor VII; F2, Prothrombin; ESR1, Estrogen receptor; NQO1, NAD(P)H dehydrogenase 1; GGCX, Vitamin K-dependent gamma-carboxylase; VKORC1, Vitamin K epoxide reductase complex subunit 1; VKORC1L1, Vitamin K epoxide reductase complex subunit 1-like protein 1; IMPDH1, Inosine-5′-monophosphate dehydrogenase 1; IMPDH2, Inosine-5′-monophosphate dehydrogenase 2.

We tried to identify the systems-pharmacological mechanism of fusarubin. The target genes of fusarubin were predicted using the BATMAN-TCM platform and related biological pathways were investigated using GSEA. The significant pathways identified from fusarubin were as follows: ubiquinone and other terpenoid-quinone biosynthesis (number of target genes: 4), complement and coagulation cascades (number of target genes: 6), drug metabolism (number of target genes: 2), purine metabolism (number of target genes: 2), and pathways in cancer (number of target genes: 3) (Figure 5).

Ubiquinone-10, also known as coenzyme Q10, is well known as a free-radical-scavenging antioxidant. Decreased levels of ubiquinone-10 are observed in many pathologies, such as neurodegenerative diseases, and Acquired Immune Deficiency Syndrome (AIDS). The reduction of ubiqinone-10 is associated with generation of free radicals and their action on cells and tissues [31,32]. Also, fusarubin has been known as inhibitor of mitochondrial NADH:ubiquinone reductase [33]. Taken together, these results suggest the possibility that fusarubin exhibits antioxidant and neuroprotective actions.

**Figure 6:**
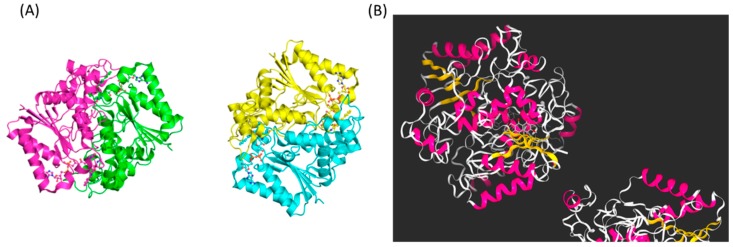
(**A**) The structure of the NQO1 protein. (**B**) Molecular docking results of fusaribin with the NQO1. This figure was created by Kdeep program (https://playmolecule.org/Kdeep/).

To further validate our systems pharmacological analysis results, we conducted molecular docking for the ligand-protein interaction using Kdeep, a 3D-convolutional neural network-based framework. Among the predicted targets of fusarubin related to ubiquinone pathway, we focused on NQO1. It was known that NQO1 converts ubiquinone into ubiquinol and performs antioxidant protection [34]. The result showed that fusarubin had a strong binding affinity to NQO1, even higher than the two previously known ligands, ARH and FAD (Figure 6 and Table 1). These results imply that fusarubin directly interacts with the NQO1, leading to the converting of the ubiquinone.

## 4. Discussion

The use of natural antioxidants for scavenging free radicals and maintaining the homeostasis contributes to alleviating neuronal dysfunction [4]. Typically, natural compounds are multiple-target molecules found mainly in microorganisms and plants and exhibit strong antioxidant activity [5,35]. Phytochemicals from natural sources also exhibit various beneficial effects in cancer, inflammation, and neurodegenerative disorders [5]. This broad spectrum of biological or pharmacological activities has made phytochemicals suitable candidates for treating multifactorial diseases, such as neurodegenerative diseases [36]. Glutamate induces oxidative stress-mediated neuronal cell death in both acute brain injuries as well as neurodegenerative diseases [37]. Oxidative stress is a major event during neuronal cell death. As a result, preventing ROS generation is a probable strategy for attenuating neuronal cell death. To assess the neuroprotective effects of isolated compounds on glutamate-induced oxidative stress, we incubated HT22 cells with 5 mM glutamate in the absence or presence of compounds for 24 h. We found that glutamate decreased the cell viability. However, treatment with the compounds 1, 4, and 6 increased cell viability significantly compared to that in the glutamate-treated cells. Among them, compound 6 (fusarubin) exerted the strongest protection effect. Morphologically, compound 6 almost completely inhibited HT22 cell death induced by glutamate. We then tested the antioxidant activities of compound 6 using a DPPH-scavenging activity assay, and the result showed that fusarubin (compound 6) exhibited a strong antioxidant property as well. Our results indicate that fusarubin markedly prevents glutamate-induced HT22 cell death, possibly through its antioxidant activity. In this study, we isolated six compounds: compounds **1** and **2** belonged to γ-pyrones, while compounds **3**–**6** belonged to naphthoquinones. Among the γ-pyrones (**1** and **2**), compound **1** was more active, indicating that oxygenation at C-13 of the side chain in the γ-pyrone is more important for the activity than the oxygenation at C-11′. As for the naphthoquinones (**4**–**6**), compound **4** was moderately active, suggesting that the hydroxyl group at the side chain of the naphthoquinone is important for the activity. Notably, compound **6** was remarkably more active than **4**. Therefore, the formation of the pyran ring together with the hydroxyl group at the ring might be critical for the activity.

We suggested the potential pathway of fusarubin using a systems-pharmacological approach and further validated the results by using molecular docking. Interestingly, the results showed that fusarubin is deeply related to vitamin K. Coagulation cascades and ubiquinone synthesis were found to be related to many vitamin K dependent targets, including Vitamin K-dependent protein S (PROS1), Vitamin K-dependent protein C (PROC), Vitamin K-dependent gamma-carboxylase (GGCX), Vitamin K epoxide reductase complex subunit 1 (VKORC1), and Vitamin K epoxide reductase complex subunit 1-like protein 1 (VKORC1L1). In addition, predicted targets associated with the vitamin K cycle were identified as being included in the gene set (GGCX, NQO1, VKORC1). Vitamin K is known to affect the generation of neuron myelin through GAS6, a vitamin K-dependent gene [38]. Vitamin K has also been studied as a growth factor for oligodendrocytes and Schwann cells [39,40]. However, pathways involving neuroprotective actions with vitamin K have not yet been established in the pathway database. Further studies may suggest the possibility of a neuroprotective pathway of vitamin K.

## Figures and Tables

**Table 1:** The binding affinity results of molecular docking. If the pK_d_ is large, then the binding affinity is increased. If ΔG is negative, then ligand and protein can be combined. K_d_, equilibrium dissociation constant; pK_d_, −log (K_d_); ΔG, Gibbs free energy; ARH, 3-(hydroxymethyl)-1-methyl-5-(2-methylaziridin-1-yl)-2-phenyl-1h-indole-4,7-dione (Pubchem CID: 5287703); FAD, Flavin-adenine dinucleotide (Pubchem CID: 643975).

Ligand Name	Molecular Weight	pK_d_	ΔG (kcal/mol)
Fusarubin	306.07	7.8	−10.53
ARH	322.13	7.55	−10.19
FAD	785.16	6.8	−9.18
